# Pu-Erh tea and GABA attenuates oxidative stress in kainic acid-induced status epilepticus

**DOI:** 10.1186/1423-0127-18-75

**Published:** 2011-10-20

**Authors:** Chien-Wei Hou

**Affiliations:** 1 Department of Biotechnology, Yuanpei University, Hsinchu, Taiwan

**Keywords:** GABA, Epilepticus, MAPKs, ROS, COX-2

## Abstract

**Background:**

Pu-Erh tea is one of the most-consumed beverages due to its taste and the anti-anxiety-producing effect of the gamma-aminobutyric acid (GABA) if contains. However the protective effects of Pu-Erh tea and its constituent, GABA to kainic acid (KA)-induced seizure have not been fully investigated.

**Methods:**

We analyzed the effect of Pu-Erh tea leaf (PETL) and GABA on KA-induced neuronal injury *in vivo *and *in vitro*.

**Results:**

PETL and GABA reduced the maximal seizure classes, predominant behavioral seizure patterns, and lipid peroxidation in male FVB mice with status epilepticus. PETL extracts and GABA were effective in protecting KA-treated PC12 cells in a dose-dependent manner and they decreased Ca^2+ ^release, ROS production and lipid peroxidation from KA-stressed PC12 cells. Western blot results revealed that mitogen-activated protein kinases (MAPKs), RhoA and cyclo-oxygenase-2 (COX-2) expression were increased in PC12 cells under KA stress, and PETL and GABA significantly reduced COX-2 and p38 MAPK expression, but not that of RhoA. Furthermore, PETL and GABA reduced PGE_2 _production from KA-induced PC12 cells.

**Conclusions:**

Taken together, PETL and GABA have neuroprotective effects against excitotoxins that may have clinical applications in epilepsy.

## Background

Pu-erh tea is one of the most widely consumed beverages in the Orient. In recent years, studies the possible investigating health benefits of Pu-erh tea have shown salutary effects on oxidative stress, cancer, cholesterol levels, blood pressure, and blood sugar, and the bacterial flora of the intestines [1-6]. Soluble ingredients in Pu-erh tea fermented with *S. bacillaris *or *S. cinereus *enhance the content of gamma-aminobutyric acid (GABA) and statin [7,8]. GABA metabolism in substantia nigra (SN) plays a key role in seizure arrest. When seizures stop, a major increase in GABA synthesis in postictal SN. GABA synthesis in SN may be reduced in status epilepticus [9]. Studies have shown that tea and its bioactive constituents may decrease the incidence of dementia, Alzheimer's disease and Parkinson's disease [10,11]; however, its effect on epilepsy has not been thoroughly investigated.

Status epilepticus (SE) is defined as a period of continuous seizure activity and has been implicated as a major predisposing factor for the development of mesial temporal sclerosis and temporal lobe epilepsy [12]. This emergency condition requires prompt and appropriate treatment to prevent brain damage and eventual death. Animal studies have shown that SE causes recurrent spontaneous seizures; i.e., epilepsy [13]. and releases free radicals from experimental models of kainic acid toxicity [14,15].

Kainic acid (KA), a glutamate-related compond, increases nerve excitability, and is widely used to induce limbic epilepsy in animal models [16]. KA causes neuron epilepticus and excitotoxicity with the increased production of reactive oxygen species (ROS) and lipid peroxidation [17-19]. Mitogen-activated protein kinases (MAPKs) and Rho kinases are associated with seizures, inflammation and apoptosis [20-22]. KA triggers neurons membrane depolarization by the release of calcium ions which are involved in nerve impulse transmission as the calcium action potential reaches the synapse [19]. A apoptosis of nerve cells can result in the release of calcium ions, and activation of calcium ion-dependent enzymes, resulting in break DNA fragments of the nerve cells with death [23].

More than one third of brain neurons use GABA for synaptic communication and the concentration of brain GABA regulates the mental and the physical health of humans [24]. GABA has been implicated in many human disease states, including anxiety and sleep disorders, epilepsy and seizures, learning and memory disorders [24-27]. Since GABA is abundant in short-term fermented Pu-erh tea [7] and has a strong antioxidant activity [28], it might protect human cells from injury by scavenging of free radicals. Therefore, the aim of this study was to investigate the protective mechanisms of GABA and Pu-erh tea leaf extract on KA-induced injury in neuronal cells *in vivo *and *in vitro*.

## Methods

### Materials

GABA and kainic acid (KA) were obtained from Sigma-Aldrich (Steinem, Germany) and Cayman Chemical (Ann Arbor, MI, USA), 2', 7'-dichlorodihydrofluorescein diacetate (H_2_DCF-DA) was obtained from Molecular Probes (Eugene, OR, USA).

### Pu-Erh tea leaf extract

Pu-Erh tea leaves were prepared as described by Hou *et al *[8]. Briefly, Pu-Erh tea leaves were ground to a fine powder with the aid of a stainless-steel mill and stored and dried to constant weight in a vacuum desiccator. With regard to the extraction procedure, triplicate one-gram samples of Pu-Erh powder from each site was mixed with 20 ml of reverse osmosis water, vortexed vigorously for 5 min, and then centrifuged at 2,000 × g for 10 min. The tea extracts were sterilized by filtration through a 0.25 μ m Millipore membrane filter (Millipore, Bedford, USA).

### Determination of GABA content

The quantity of GABA in extracts of Pu-Erh tea was determined using the method described by Zhang and Bown [29]. Tea liquor was prepared as described above with 200 mg of dry tea powder. Samples of standard tea liquor (1 mL each) were placed in glass tubes to which was added 0.6 mL of 0.1 M lysis buffer and 1 mL of 0.3% 2-hydroxynaphthaldehyde (the derivatizing reagent) (TCI, Japan). The tubes were placed in a water bath for 10 min maintained at 80°C and then cooled to room temperature. Sufficient methanol was then added to give a final volume of 5 mL. The guard and analytical column used in HPLC analysis was Merck LiChrosper100 RP18 (5 μ m, 4.0 mm i.d. × 15 cm). The mobile phase was comprised of methanol and H_2_O (62:38), the flow speed was 1.0 mL/min, the detection wavelength was 330 nm, and the injection amount was 20 μ L. GABA standard liquor was prepared by diluting GABA with pure water to different strengths (10, 50, 100, 150, and 200 μ g/mL) to obtain different chroma values. The derivatization reaction was observed with GABA liquor at five values of chroma. Each sample was tested three times, and the average value of the absorbance at different values of concentration was calculated.

### Oxidative stress in mice

Adult male FVB mice, body weight 30-35 g, were used for this experiment. SE was induced by KA (10 mg/ml in phosphate-buffered saline (PBS), 10 mg/kg, subcutaneous injection). Pu-Erh tea leaf (PETL) powder and GABA was separately diluted in normal saline 10 mg/ml and 1 mg/ml. The animals were fed with PETL (10 mg/kg) and GABA by gavage for 3 days before the KA experiment. The control group was fed with an equal volume of vehicle (normal saline). The procedures were conducted in accordance with the Taichung Veterans General Hospital Animal Care and Use Committee, Taichung, Taiwan (IACUC Approval No. LA-99741) and all possible steps were taken to avoid animals' suffering at each stage of the experiment. Diazepam at lethal dosage, 60 mg/kg i.p., was given to stop seizures 2 h after KA injection and the animals were sacrificed by decapitation under CO_2 _asphyxia. The whole brain was immediately removed and frozen in liquid nitrogen and stored at -70°C until use.

Malondialdehyde (MDA), a thiobarbituric acid reacting substance (TBARS) was used as an indicator of lipid peroxidation. To estimate oxidative stress, the amount of TBARS in the brain from each group was measured. Manual homogenization of brains was carried out at 4°C using cold lysis buffer. Protein concentration of the homogenate was determined by BCA protein assay using bovine serum albumin as a standard. For TBARS assay [30], the sample (0.2 ml) was mixed with the same volume of 20% (w/v) trichloroacetic acid (TCA) and 1% (w/v) thiobarbituric acid in 0.3% (w/v) NaOH. The mixture was heated in a water bath at 95°C for 40 min, cooled to room temperature and centrifugated at 5000 rpm for 5 min at 4°C. The fluorescence of the supernatant was determined by spectrophotometry with excitation at 544 nm and emission at 590 nm.

### Mortality and behavior

Mice were fed with and without PETL extract or GABA for 3 days before the SE experiment was conducted. The control group was treated with the vehicle (normal saline). SE was induced with kainic acid (KA, 10 mg/kg, s.c.). Each behavioral seizure was recorded according to a modification of the classification from Racine [31]: 0, exploring; 1, immobility 2, rigid posture; 3, head nodding; 4, bilateral forelimb clonus and falling; 5, continued clonus and falling; 6, generalized tonus. Three behavioral patterns of SE could be recognized: I, initial (class 1-2), M, middle (class 3) and C, critical (class 4-6). Diazepam, 25 mg/kg i.p., was given to stop seizures at 5 hours of SE and the 10-h mortality rate was recorded.

### TUNEL Staining

Adult male FVB mice were observed and recorded the behavior of status epilepticus severity induced by KA stress. After recovery for 24 h, mice were injected with a lethal intraperitoneal injection of pentobarbital (120 mg/kg), and brain tissue sections were perfused with 4% paraformaldehyde for fixation. Coronal paraffin sections were prepared with Hematoxylin and Eosin (H&E) staining for cells damage and TUNEL staining to assess apoptosis study. After fixation for 1 h, mice brain sections were added with freshly prepared permeabilisation solution (0.1% (v/v) Triton X-100 in 0.1% sodium citrate) and then washed with cold PBS and added with TUNEL stain mixture (Roche, Mannheim, Germany), at 37°C in the dark, for 1 h. The apoptosis of neuronal cells was quantified by fluorescence microscopy with excitation at 450-500 nm and detection wavelength at 515-565 nm.

### Cell culture

The Rat pheochromacytoma cell line PC12 was maintained in Dulbecco's modified Eagle's medium (DMEM) supplemented with 10% (v/v) fetal bovine serum, 5% horse serum, 100 U/ml penicillin and 100 μ g/ml streptomycin at 37°C in a humidified incubator under 5% CO_2_. Confluent cultures were passaged by trypsinization. Cells were washed twice with warm DMEM (without phenol red), then treated in serum-free medium. In all experiments, cells were treated with GABA and/or KA-stress for the indicated times.

### Preparation of cell extracts

Test medium was removed from culture dishes and cells were washed twice with ice-cold phosphate-buffered saline, scraped off with the aid of a rubber policeman, and centrifuged at 200 × g for 10 min at 4°C. The cell pellets were resuspended in an appropriate volume (4 × 10^7 ^cells/ml) of lysis buffer containing 20 mM Tris-HCl, pH 7.5, 137 mM NaCl, 10 μ g/ml aprotinin, and 5 μ g/ml pepstain A. The suspension was then sonicated. Protein concentration was determined by Bradford assay (Bio-Rad, Hemel, Hempstead, UK) after cells were suspended to 2 mg/ml with in lysis buffer.

### Western blotting

Protein samples containing 50 μ g of protein were separated on 12% sodium dodecyl sulfate polyacrylamide gels and transferred to Immobile polyvinylidene difluoride membranes (Millipore, Bedford, MA, USA). Membranes were incubated for 1 h with 5% dry skim milk in TBST buffer (0.1 M Tris-HCl, pH 7.4, 0.9% NaCl, 0.1% Tween-20) to block nonspecific binding, and then incubated with rabbit anti-COX-2, Rho A (1:1000; Cayman chemical; Cell Signaling, USA), and anti-phospho-MAPKs. Subsequently, membranes were incubated with secondary antibody streptavidin-horseradish peroxidase conjugated affinity goat anti-rabbit IgG (Jackson, West Grove, PA, USA).

### Reactive oxygen species generation

Intracellular accumulation of ROS was determined using H_2_DCF-DA, which is a nonfluorescent compound that accumulates in cells following deacetylation. H_2_DCF then reacts with ROS to form fluorescent dichlorofluorescein (DCF). PC12 cells were plated in 96-well plates and grown for 24 h before addition of DMEM plus 10 μM H_2_DCF-DA, incubaed for 60 min at 37°C, and treated with 150 μM KA for 60 or 120 min. Cells were then washed twice at room temperature with Hank's balanced salt solution (HBSS without phenol red). Cellular fluorescence was monitored on a Fluoroskan Ascent fluorometer (Labsystems Oy, Helsinki, Finland) using an excitation wavelength of 485 nm and emission wavelength of 538 nm.

### MTT reduction assay for cell viability

Cell viability was measured using blue formazan that was metabolized from colorless 3-(4,5-dimethyl-thiazol-2-yl)-2,5-diphenyl tetrazolium bromide (MTT) by mitochondrial dehydrogenases, which are active only in live cells. PC12 cells were preincubated in 24-well plates at a density of 5 × 10^5 ^cells per well for 24 h. Cells incubated with various concentrations of GABA were treated with 150 μM KA for 24 h, and grown in 0.5 mg/ml MTT at 37°C. One hour later, 200 μ l of solubilization solution was added to each well and absorption values read at 540 nm on microtiter plate reader (Molecular Devices, Sunnyvale, CA, USA). Data were expressed as the mean percent of viable cells vs. control.

### Lactate dehydrogenase (LDH) release assay

Cytotoxicity was determined by measuring the release of LDH. PC12 cells treated with various concentrations of GABA were incubated with 150 μM KA for 24 h and the supernatant was then assayed for LDH activity. A absorbance was read at 490/630 nm using a microtiter plate reader. Data were expressed as the mean percent of viable cells vs. 150 μM KA control.

### Calcium release assay

PC12 cells with various concentrations of GABA were treated with 150 μM KA for 24 h and the supernatant was used to assay the release of Ca^2+^. The 10 μ l supernatant was added to 1 ml Ca^2+ ^reagent (Diagnostic Systems, Holzheim, Germany) and mixed well, allowed to stand for 5 min, then transferred the 100 μ l supernatant to 96 well. Calcium concentration was determined using a microplate reader with a 620 nm absorbance and quantified with a 10 mg/ml Ca^2+ ^standard solution.

### Measurement of lipid peroxidation

Lipid peroxidation was assessed by measuring malondialdehyde (MDA) in extracts of PC12 cells using a lipid peroxidation assay kit (Cayman Chemical, Ann Arbor, MI, USA). This kit works on the principle of condensation of one molecule of either malondialdehyde (MDA) or 4-hydroxyalkenals with two molecules of N-methyl-2-phenylindole to yield a stable chromophore. MDA levels were assayed by measuring the amount produced by 5 × 10^5 ^cells. A absorbance at 500 nm was determined using an ELISA reader (spectraMAX 340, Molecular Devices, Sunnyvale, CA, USA).

### Assay of PGE_2 _concentration and Caspase-3 Activation

PGE_2 _release and caspase-3 activity were measured by ELISA assay. PC12 cells (5 × 10^5^) were added to 0.5 ml homogenization buffer (0.1 M phosphate pH 7.4, 1 mM EDTA) and homogenized. The lysate was then centrifuged at 12,000 × g for 15 min at 4°C. The supernatant was transferred to a clean test tube, and its total protein content was analyzed using the Bradford assay (Bio-Rad, Hemel, Hempstead, UK). PGE_2 _concentration and caspase-3 activity were determined using PGE_2 _and caspase-3 ELISA kits (R&D Systems, Minneapolis, MN, USA). A absorbance at 450 nm was determined using a microplate reader (spectraMAX 340, Molecular Devices, Sunnyvale, CA, USA).

### Statistical analysis

All data were expressed as the mean SEM. For single variable comparisons, Student's test was used. For multiple variable comparisons, data were analyzed by one-way analysis of variance (ANOVA) followed by Scheffe's test. P values less than 0.05 were considered significant.

## Results and discussion

We analyzed short-term fermented Pu-erh tea samples processed with tea-leaf extract for the content of GABA [28]. The amount of the bioactive component GABA in the Pu-erh tea leaf was 177 ± 35 μ g/g.

### Effect on mortality and behavior

Treatment of FVB mice with PETL or GABA on KA-induced SE did not affect mortality (Table 1). However, PETL and GABA both significantly attenuated the maximal seizure classes and the predominant behavioral seizure patterns in the SE mice compared with the vehicle (Table 1, GTL and GABA, p < 0.001,).

**Table 1:** Effects of Pu-Erh tea leaf extract and GABA on the predominant behavior patterns/maximal seizure class (MSC) and 10-h mortality rate of the mice with 5-hour KA-induced SE

Variables	V-10	PETL-10	*p-*value	GABA-1	*p-*value
	n (%)	n (%)		n (%)	
**Mortality**	0 (0)	0 (0)	0.000^a^	0 (0)	0.000^a^
**Behavior Pattern/MSC**					
**I/class 1-2**	0 (0)	0 (0)	0.000^b^	0 (0)	0.000^b^
**M/class 3**	2 (17)	10 (83)	0.000^c^	12 (100)	0.000^c^
**C/class 4-6**	10 (83)	2 (17)		0 (0)	

^a^Fisher's exact test.

^b^Pearson's chi-square test: all seizure classes taken together.

^c^Kendall's tau-c: all seizure classes taken together.

I: Initial (class 1-2). M: middle (class 3). C: critical.

PETL-10: Pu-Erh Leaf extract, 10 mg/kg.

GABA-1: gamma-aminobutyric acid, 1 mg/kg.

V-10: vehicle control, with normal saline.

### Protection from KA toxicity

**Figure 1:**
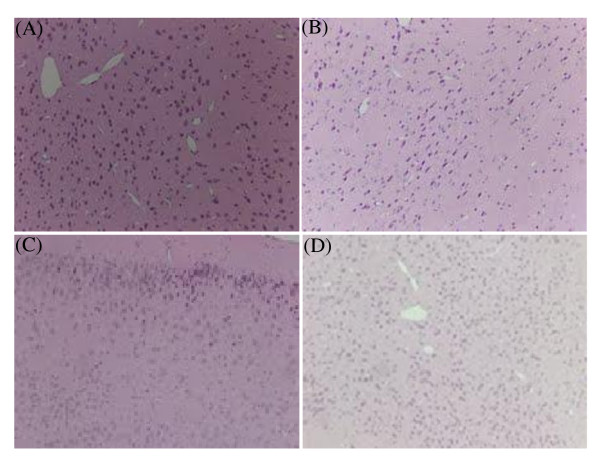
**H&E stain of KA-stressed FVB mice cortex. Kainic acid (KA, 10 mg/kg) caused neuronal damage**. After 5 h KA-induced SE of FVB mice, the cortex was observed with cell shrinkage and long shape (B). PETL 10 mg/kg (C) or GABA 1 mg/kg (D) significantly reduced KA-induced neuronal damage in cortex of the FVB mice as compared to control (A). (20x)

**Figure 2:**
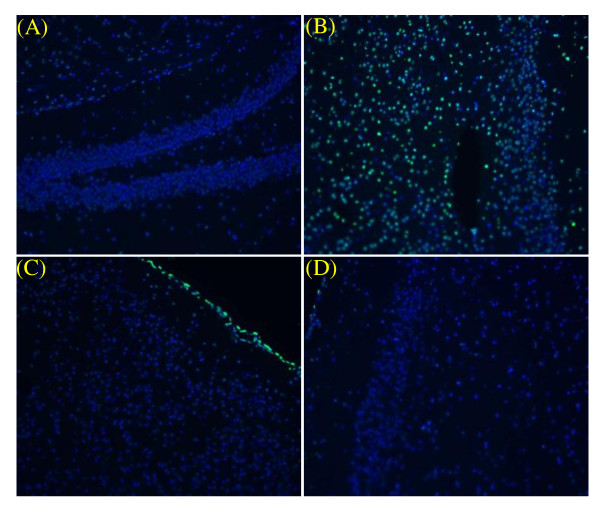
**DAPI and TUNEL staining of hippocampus form KA-stressed mice**. KA induced apoptosis (green fluorescence) of hippocampus neurons on vehicle control mice (B). The TUNEL staining showed that 10 mg/kg PETL (C) and 1 mg/kg GABA (D) significantly reduced KA-induced apoptosis in hippocampus of the FVB mice brain as compared to control (A). (200x)

**Figure 3:**
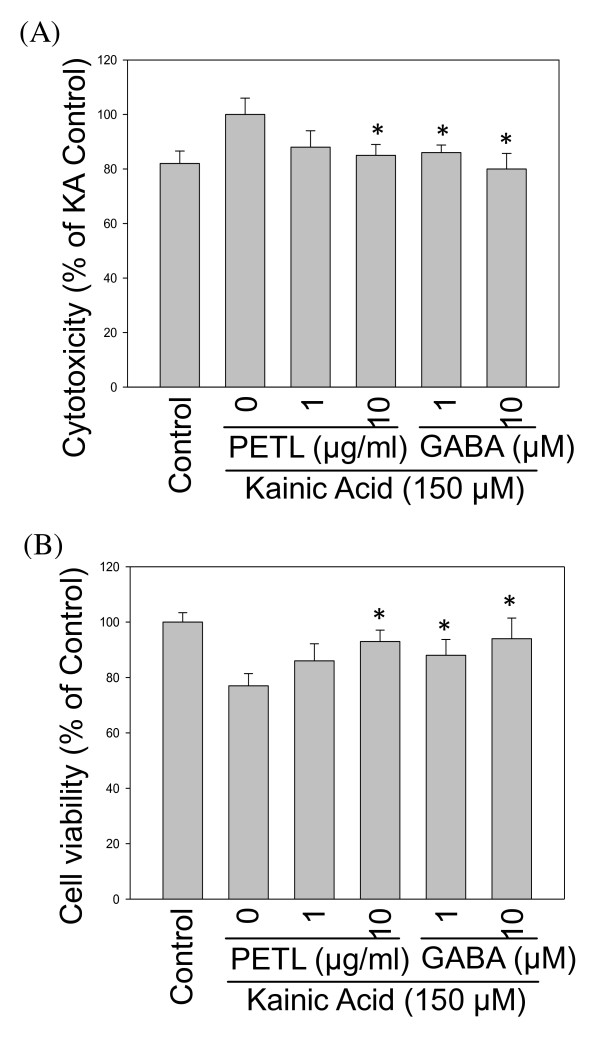
**Effect of PETL extract and GABA on cell viability and cytotoxicity of KA-stressed PC12 cells**. Cells were treated with KA (150 μM) alone or with various concentrations of PETL extract (1, 10 μ g/ml) or GABA (0.1, 1, 10 μM) for 24 h. LDH (A) release was decreased and cell viability (B) was increased by PETL extract and GABA. *P < 0.01 as compared to KA control.

We further evaluated H&E stained section of the brains of KA-stressed FVB mice. KA (10 mg/kg) caused epilepticus and neuronal damage. However, after PETL (10 mg/kg) or GABA (1 mg/kg) treatment, the damage in cortical neuronal cells was reduced (Figure 1). The TUNEL staining assay showed that PETL or GABA significantly reduced KA-induced apoptosis in hippocampus of the FVB mice as compared to the control (Figure 2). In order to understand the protective mechanism, KA-induced injury in neuronal PC12 cells were investigated using LDH and the MTT assay. As shown in Figure 3, PC12 cells were protected from the injury by the PETL extract (1, 10 μ g/ml) and GABA (0.1, 1, 10 μM). The reduction in LDH release and increase in cell viability caused by the PETL extract and GABA were consistent with the *in vivo *data.

### KA-induced calcium release

**Figure 4:**
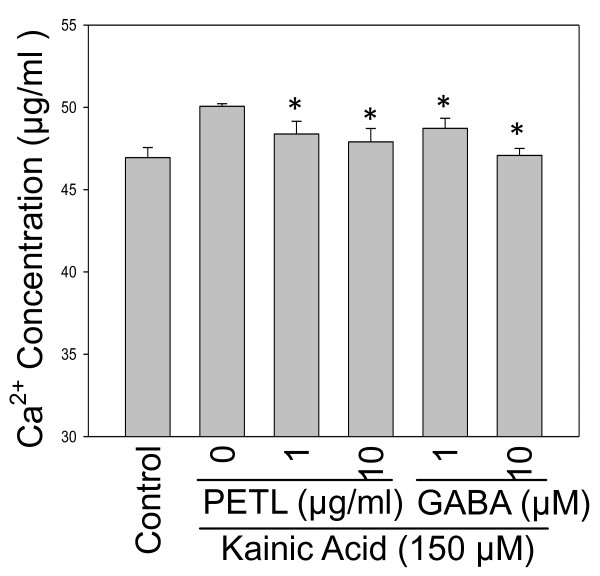
**Effect of PETL extract and GABA on Ca^2+ ^generation from KA-treated PC12 cells**. Cells were treated with KA (150 μM) alone or with various concentrations of PETL extract or GABA for 24 h. PETL and GABA were effectively reducing the release of Ca^2+ ^under KA stress. *P < 0.01 as compared to the KA control.

KA triggers neuronal membrane depolarization by releasing calcium ions from neuron cells [32]. In the present study, KA induced calcium release from PC12 cells in a time-dependent manner (data not show). PETL extract and GABA significantly reduced KA-induced calcium release in PC12 cells (Figure 4).

### ROS and lipid peroxidation

**Figure 5:**
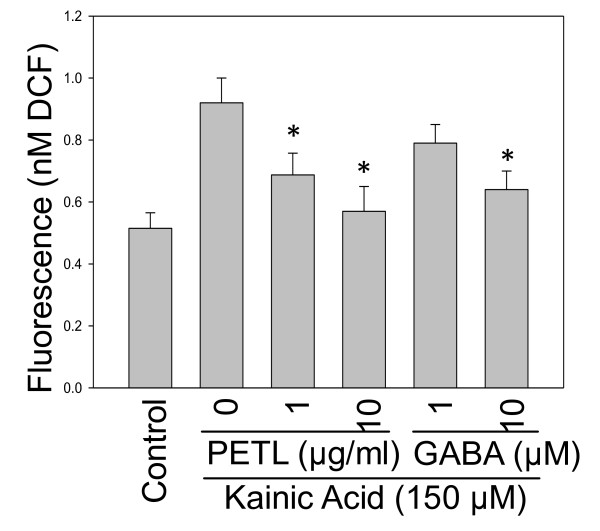
**Effect of PETL extract and GABA on ROS generation in PC12 cells under KA stress**. PETL extract (1, 10 (j,g/ml) and GABA (0.1, 1, 10 uM) were effectively reducing the ROS production from PC12 cells induced by KA stress (150 uM) at 120-min. *P < 0.01 as compared to the KA control.

**Figure 6:**
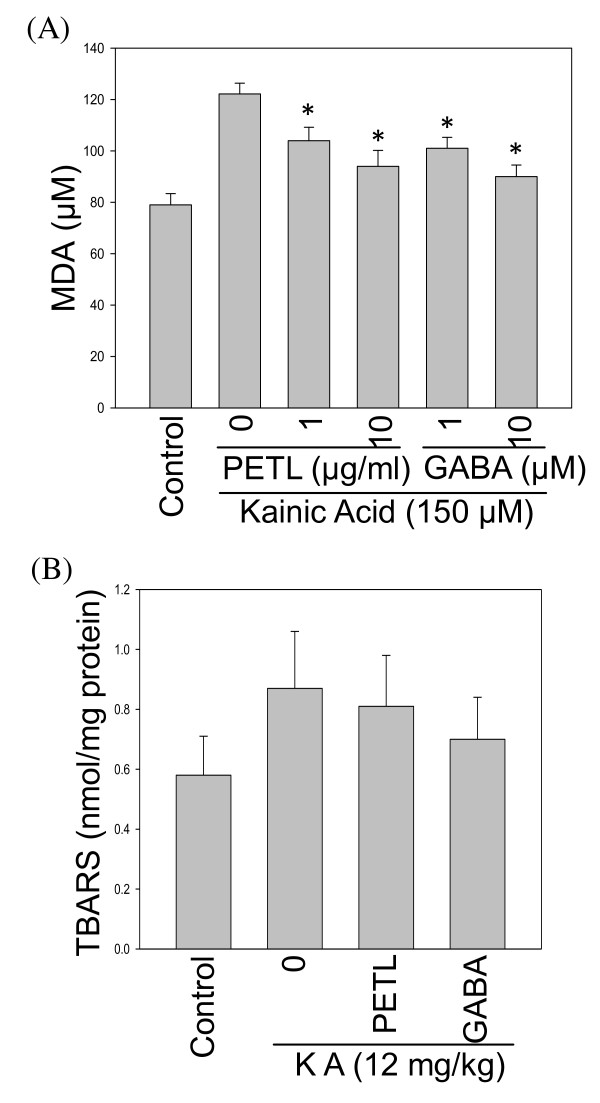
**In vitro and in vivo effect of PETL extract and GABA on the KA-induced oxidative stress**. KA-induced lipid peroxidation of PC12 cells and brain neuron tissue of FVB mice were determined by ELISA and spectrophotometry, respectively. PETL or GABA was effectively reducing lipid peroxidation of PC12 cells by under 24-h KA stress (A) and in mice with 2-h KA-induced SE (B). *P < 0.01 as compared to the KA control.

ROS and lipid peroxidation can damage neuronal cells [16,18]. KA-treated cells increased DCF fluorescence by 80% after 120 min as compared with the control cells. Treatment with PETL extract or GABA protected cells against KA cytotoxicity by decreasing KA-induced ROS accumulation (Figure 5). Marked increases in MDA and 4-hydroxyalkenals levels were observed in KA-exposed cells, as compared with the control cells (Figure 6A). The PETL extract and GABA significantly protected cells against KA toxicity by lowering MDA levels (p < 0.01, as compared to the KA-treated cells). PETL and GABA were Consistently effective in reducing TBARS levels in the KA-induced SE mice (Figure 6B, P < 0.01 as compared to the KA control).

### Caspase-3 activation

**Figure 7:**
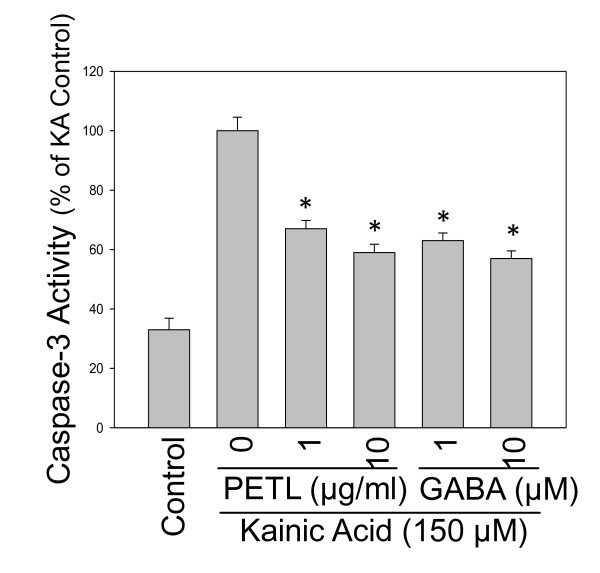
**Kainic acid-induced caspase-3 activation**. Cells were treated with KA (150 μM) alone or with PETL extract and GABA in various concentrations for 24 h. Both PETL and GABA decreased the caspase-3 activity significantly, *P < 0.01 as compared to the KA control.

Status epilepticus causes the death of nerve cells partly due to apoptosis. PETL and GABA significantly reduced KA-induced apoptosis in hippocampus cells of the mice (Figure 2). Therefore, we further evaluated whether the apoptotic signaling pathways was involved in the KA-treated PC12 cells. KA and GABA affected caspase-3 activation (Figure 7). Cells were treated with KA (150 μM) alone or with PETL extract or GABA in various concentrations for 24 h. Both PETL and GABA decreased the caspase-3 activity significantly in KA-treated PC12 cells.

### COX-2 and MAPKs activation

**Figure 8:**
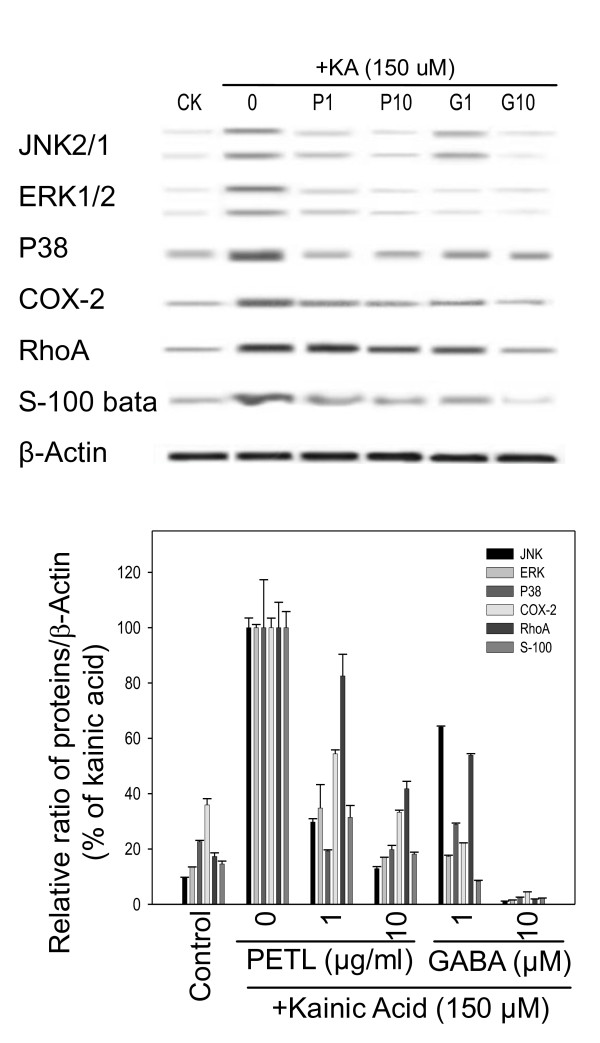
**Effect of PETL extract and GABA on KA-activated signaling pathway**. COX-2, JNK, ERK, p38 MAP kinases, and RhoA in PC12 cell under KA stress for 30-min was determined by Western blot assay. Values represent the mean from three independent experiments. *P < 0.05 as compared to the KA control.

The effect of GABA or PETL extract on KA-induced signaling pathways in PC12 cells was evaluated by Western blot assay. KA induced the cell signal activation of MAP kinases (JNK, ERK. P38), COX-2, RhoA, and S100 in PC12 cells at 30 min. Only the activated COX-2 and MAPKs expression, but not RhoA were suppressed by GABA and PETL extract as compared to KA controls. GABA suppressed 50~80% COX-2 expression whereas GABA and PETL suppressed 80~90% S100-beta expression level as compared to KA controls (Figure 8).

### Effect of GABA on PGE_2 _production in PC12 cells

**Figure 9:**
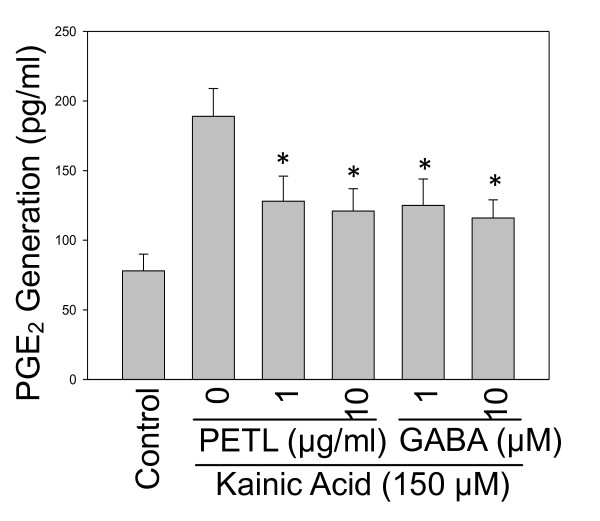
**Effect of PETL extract and GABA on PGE_2 _production**. PETL extract and GABA, significantly reduced the PGE_2 _production of KA-induced PC12 cells. *P < 0.01 as compared to the KA control.

Since COX-2 controls PGE_2 _production, we inquired whether KA-induced COX-2 would affect PGE_2 _production. We found that PETL extracts and GABA significantly reduced the PGE_2 _production in KA-induced PC12 cells as predicted. PETL extracts and GABA reduced 30~40% PGE_2 _production as compared with the KA control cells. (Figure 9).

## Discussion

The main result of the present study is the finding PETL and GABA protected animals from KA-induced brain injury. MDA and apoptosis were significantly reduced in the GABA and PETL-treated animals as compared with the vehicle control (Figure 2 and Figure 6). This effect was confirmed by the *in vitro *effects of GABA and PETL: decreased LDH release, ROS generation, lipid peroxidation, caspase-3 activation, and the increased cell viability of KA-stimulated PC12 cells. GABA appears to be a well bioactive component in the extract of Pu-Erh tea leaves. GABA has long been advocated for the treatment of cancer, oxidative stress, inflammation and diabetes, but few studies have evaluated modes of action in these processes. The present study demonstrates that GABA was effective in protecting PC12 cells from KA-induced injury in a dose-dependent manner. GABA and PETL extract decreased KA-induced Ca^2+ ^and ROS release and lipid peroxidation in PC12 cells and FVB mice. Western blot analysis revealed that MAPKs, COX-2, RhoA and S-100 expression were increased in PC12 cells under KA stress. However, MAPKJNK2/1, MAPKERK1/2, COX-2 and RhoA expression but not MAPK p38 were significantly reduced by GABA (10 μM). Furthermore, GABA and PETL treatment reduced PGE_2 _production by PC12 cell under KA stress.

PC12 cells derived from rat pheochromacytoma have been widely used for neurological studies [33,34]. Increases in ROS accumulation and lipid peroxidation were observed in KA-treated PC12 cells. KA-induced ROS accumulation was significantly reduced by PETL extract or GABA (Figure 4). These observations agree with earlier reports that shown that kainate induces lipid peroxidation in the rat neurons [14,35]. Lipid peroxidation is essential to assess the role of oxidative injury in pathophysiological disorders [36,37]. Lipid peroxidation results in the formation of highly reactive and unstable hydroperoxides of saturated or unsaturated lipids. We found that KA induced the activation of MAP kinases (JNK, ERK, p38), RhoA, S100, and COX-2 in PC12 cells. It is noteworthy that KA-activated COX-2 and MAPKs were reduced by GABA and PETL extract. In particular, GABA suppressed KA-activated S100, COX-2 and MAPKs expression. This result is in accord with observation that administration of tea extract (TF3) to rats with cerebral ischemia-reperfusion reduced mRNA and protein expression of COX-2, iNOS and NF-κB activation in treated animals [38]. *In vitro *studies showed that antioxidants suppress PGE_2 _production and COX-2 activity in lipopolysaccharide (LPS)-activated macrophages and microglia cells [39,40]. Consistently, Icariin attenuates lipopolysaccharide-induced microglial activation and resultant death of neurons by inhibiting TAK1/IKK/NF-κB and JNK/p38 MAPK pathways [40]. The present results are consistent with previous reports which show that KA-induced neuronal death can be prevented either by inhibiting xanthine oxidase, a cellular source of superoxide anions, or by the addition of free radical scavengers to the culture medium [41]. ROS generation is correlated with KA induced-excitotoxicity [16,18,41,42]. The ability of kainate to induce lipid peroxidation is also related to the exposure of excitotoxin to the brain [42]. It is widely accepted that neuronal degeneration after KA administration is associated with a depletion of AT P and accumulation of [Ca^2+^]i in neuron. The increase in [Ca^2+^]i can trigger Ca^2+^-activated free radicals formation [41]. Thus, our data showing suppression of ROS and Ca^2+ ^release by PETL are consistent with the proposed role of GABA and PETL extract in neuronal protection.

Cytokines and chemokines play key roles in the inflammatory response and its perpetuation [43,44]. It is conceivable that besides factors canonically implicated in the inflammatory response, other factors, including members of the S100 protein family [45,46], act to sustain the inflammatory response or to determine direct effects on neurons and/or microglia, thus switching the inflammatory response to neuronal death. The Ca^2+^-modulated protein of S100B is thought to be one factor that plays such a dual role [45,46]. A role of cerebral COX-2 mRNA and protein in KA toxicity has also been postulated [47-49]. KA-induced COX-2 expression parallels the appearance of neuronal apoptotic features [47]. The KA-inducted COX-2 is also involved with free radicals formation [50]. Several protease families have been implicated in apoptosis, the most prominent being caspases [51]. However, we did find that KA affected the caspase-3 activation in PC12 cells. Since S100 and COX-2 may be involved in pathways leading to neuronal death, these additional effects of GABA could account for its neuroprotective properties, such as inhibition of KA-induced inflammatory mediators [50]. Since PGE2 was synthesised in response to activation of COX-2 expressing cells, directly hyperpolarises GABA-induced neurons [52]. GABA and PETL extract, as predicted, reduced PGE_2 _production dose-dependently, and S100, and COX-2 activation in KA-induced PC12 cells. Taken together, these results indicate that antioxidant and anti-inflammatory effects might account for the protective mechanisms of gallic acid on KA-induced PC12 cell injury.

Present data also showed that GABA or PETL could decrease the severity of seizure behavior. Further studies are needed to confirm whether GABA has direct effects on the seizure behavior and the related molecular mechanism in this issue. The present results are consistent with previous reports which show that antioxidants such as resveratrol [13] and vitamin E [53] are also protective against various animal models of SE in terms of the oxidative stress or convulsions. Resveratrol protects against KA-induced neuronal damage and subsequent epilepsy [54]. Stopping seizure activity promptly is the best way to prevent SE-induced free radical formation and neuronal damage. However, clinical experience shows that SE can be refractory to the commonly used medications. Therefore, intervention by antioxidants can be a potential beneficial approach in the treatment of SE.

## Conclusions

In conclusion, we found that Pu-Erh tea leaves had abundant content of GABA as bioactive components. The metabolites of GABA are also potent antioxidants and anti-inflammatory agents. This suggests that natural antioxidants play an important role in neuroprotection under excitotoxins and GABA in the Pu-Erh tea was responsible for this protection. Pu-Erh leaf extract and GABA ameliorates oxidative stress in KA-induced status epilepticus. The molecular mechanisms of PETL extract and GABA on SE-induced excitotoxicity warrants further study for their therapeutic potential.

**The author has no competing interests in this manuscript**.
